# Envisioning the Role of Educators’ Technological Pedagogical and Content Knowledge and Self-Regulated Learning in an English as a Foreign Language Context

**DOI:** 10.3389/fpsyg.2022.943072

**Published:** 2022-06-29

**Authors:** Wenjie Li

**Affiliations:** Department of Foreign Languages, Taiyuan University, Taiyuan, China

**Keywords:** EFL setting, self-regulated instruction, technological pedagogical and content knowledge, English as a foreign language context, education

## Abstract

In recent decades, more and more research has been conducted on the competencies of educators in improving the role of technology in academic activities. These competencies are based on a clear platform of technological knowledge, together with the recognized aspects of vast pedagogical knowledge and rich content knowledge. In such a modern era, the knowledge of technology, pedagogy, and content knowledge (TPACK) is quite vital in getting the educators ready to turn into qualified educators to cope with the difficulties of the 21st-century. Moreover, it is considered that self-regulation is a significant factor in English as a Foreign Language (EFL) educators’ learning and professional development. In line with the literature, nurturing and boosting self-regulated learning (SRL) capabilities assist individuals to gain complicated knowledge and manage challenging problems in the process of teaching. Consequently, this review makes an effort to inspect teachers’ TPACK growth by enhancing their SRL capabilities in the process of technology-based instruction. In a nutshell, the implications of the present review were presented together with suggestions.

## Introduction

Nowadays, in educational practices, using technology successfully in education becomes solely possible when educators, who are in charge of education, guidance, and direction of learners, are trained or equipped well to employ educational technologies efficiently (Önal and Çakır, 2015). The lack of knowledge, skills, and competencies of educators in the practice of technology in the education progression has been recognized as the main obstacle to the incorporation of technology (Bingimlas, 2009; Kaeophanuek et al., 2019). Educator training is developed to provide educators with the pedagogical content knowledge, competencies, and viewpoints needed for class instruction. Generally, educators have the instructional knowledge that comes from a mixture of pedagogical knowledge, content knowledge, knowledge of learners, and knowledge of ecological settings (Koehler and Mishra, 2009). With the increasing usage of technology in the academic setting, information and communication technology (ICT) has turned into a strong instrument for expanding academic chances (Tantrarungroj and Suwannatthachote, 2013). The teachers should prepare themselves with the knowledge and technological competencies and use technologies for generating more effective instruction and learning procedures (Chai et al., 2011). Technology affords the chances for the educators to state or construct a diversity of conditions and learning settings in instruction and learning procedures. With all the possibilities offered by the use of technology, there are unresolved challenges that somewhat prevent educators from incorporating the use of technology and following traditional or pedagogical methods instead (Kasim and Singh, 2017; Aghaei et al., 2020).

One cannot overemphasize the status of technology for EFL educators and learners in the process of education and scholars have emphasized the significance of the effective use of technology in education and learning (Derakhshan et al., 2021). By using technology, learners’ scientific research and reasoning might be constructively developed to assist them link constructed knowledge to practical activity. Furthermore, utilizing technology can assist the enhancement of educators’ confidence, attitudes, and instruction, and assist EFL educators to comprehend scientific notions and innovation (Lajoie and Poitras, 2017). Moreover, the incorporation of technology in education makes the education and learning process activity-centered, and learner-centered which finally enhances the development of 21st-century abilities that are much required to endure the difficulties in the upcoming knowledge communities (Garba and Alademerin, 2014). As a result, the knowledge of educators to incorporate content, education, and technology has become significant.

Educator training in the academic usage of technology seems to be an important element in every significant plan for teaching (Angeli and Valanides, 2009). One of the approaches for techno-pedagogical incorporation in the arena of technological incorporation in academic activities is the structure of technological pedagogical and content knowledge (TPACK) (Lukłianchuk et al., 2021; Smyrnova et al., 2021). The TPACK concept refers to merging the three columns of knowledge: technology, education, and material that is extensively admitted by scholars and academic specialists as a hypothetical structure and directive for educators’ professional development.

Concerning educator technology teaching, Mishra and Koehler (2006) believe that TPACK has a significant role in educators’ influential usage of academic technology for instruction. In addition, it acts as an experiential hypothetical frame to teach educators how to mix their vast technological knowledge with their pedagogical knowledge and content to give more concreteness to abstract topics in order to help learners construct new knowledge (Chai et al., 2013). The TPACK is a knowledge framework that enables educators to implement efficient learning. Educators need to learn the manner of teaching particular notions using technology (Terpstra, 2015; Tzavara and Komis, 2015). The TPACK allows educators to comprehend the features of the topic and learners, so they may select the proper learning tactics and use technology in learning. The TPACK aims at consolidating the multidisciplinary expert knowledge about Technology, Pedagogy, and Content that educators require; therefore, they can educate, and learners may learn to use technology instruments (Angeli and Valanides, 2009). Based on TPACK, using technology instruments refers to something beyond having access to the instruments and learning the technical proficiencies to manage them. Educators need to carefully think about the perspective of technology to investigate the pedagogical subjects in lesson development. It refers to deciding about choosing, adjusting, and implementing proper content, pedagogy, and technology such that it can considerably enhance the value of education with technology in the class, by using pedagogies that advocate learner-oriented learning (Angeli and Valanides, 2009; Graham, 2011). According to Magliaro and Ezeife (2007), new educators mostly have constrained knowledge about how to merge information technology into their expert activity and syllabus even though pre-service educators have formal education in educational technology. But, developing TPACK is complicated because educators must take into account learners’ particular needs as well as the limitations of learning environments when making technology-merged practices (Angeli and Valanides, 2009). Based on the literature review, Scholars have investigated various education tactics to assist teachers in merging technology into their activities. The education approach needs learners to involve in independent learning activities, where they must supervise their advancement, adopt tactics, and systematically think about their learning outcomes. Scholars identified these abilities as self-regulated learning (Levin and Wadmany, 2008) that involves using instruments, methods, or procedures that are proper to academic technology in helping the use of senses, memory, and perception to enhance educational activities and learning results (Padmavathi, 2017). Such self-regulation is taken into account as the educators’ capability to control their successes and measures, set goals for themselves, assess their achievement when they attain these goals, and compensate themselves for gaining such targets (Orbell and Alison Phillips, 2019).

Academic psychologists characterize SRL as a dynamic educational phase in which students utilize a vast scope of intellectual techniques to attain subjects and metacognitive techniques to oversee and regulate the whole cycle and SRL is central for studying intricate subjects such as science, medicine, or history (Azevedo et al., 2004; Greene and Jones, 2020). Correspondingly, scholars have commenced managing the emergent attention to people’s self-regulation in learning as a means to improve educational results (Pintrich, 2000; Zimmerman, 2008). Self-regulated students are noble tactic users who plan, set targets, choose a tactic, organize, self-supervise, and self-assess at different points during the acquisition process (Gestsdottir et al., 2011). Self-regulation refers to the ability of people to adapt to diverse conditions for developing a healthy lifestyle (Gestsdottir et al., 2011). It is argued that teacher training should not be confined to the transmission of content knowledge and pedagogical content knowledge using static approaches, but rather they should find methods and techniques for educators to build knowledge through their SRL (Michalsky and Schechter, 2013).

Self-regulation is highly potential to improve educators’ TPACK because it assists their education and organization of capabilities and properties in education (Tantrarungroj and Suwannatthachote, 2013). Self-regulated educators dynamically and practically take part in learning by shaping the information available from the peripheral setting like the class, and the internal setting like their awareness, for setting targets, enacting tactics, and producing individual meanings (Pintrich, 2000). Based on the previous studies, educators need to concentrate on their SRL abilities, since it enables them to think more profoundly about their educational activities, which may result in enhanced learner performance (Xiao et al., 2005). It is stated that educators have to be self-regulated students themselves because of constantly changing curricular modifications, which need innovation and compatibility (Delfino et al., 2010). Multiple empirical investigations exist on the function of educators’ SRL in TPACK. For instance, pre-service educators who were assisted by SRL prompts had a better performance compared to those without SRL scaffolds (Kramarski and Michalsky, 2009). In the same vein, the findings of the study by Poitras et al. (2018) indicated a stronger association between educators’ information-seeking effectiveness and their self-regulatory attempts in the situation of SRL-scaffolded TPACK development. However, to date, in china, no particular research exists on self-regulation and its correlation with TPACK of EFL educators; therefore, the current study may be a reference for educators to reinforce their self-regulation so that they can grow their abilities in the TRACK that cause higher quality learning.

Educators have problems in achieving TPACK (Kohen and Kramarski, 2012; Poitras et al., 2017) and one description is that educators have lower competency in SRL capabilities that account for educators’ dynamic control in their learning procedure by assessing and managing their attempts to get TPACK and practically apply it (Poitras et al., 2017). Nevertheless, based on the researcher’s knowledge, it is not till previous years that researchers begin to explore how self-regulation impacts EFL educators’ knowledge and professional development (Jang and Chen, 2017). However, it is required to scrutinize EFL educators’ self-regulatory cycles to reach a comprehension of its relationships with TPACK.

## Review of Literature

### Technological Pedagogical and Content Knowledge

Technological pedagogical and content knowledge alludes to educators’ successful use of technology for instruction and learning objectives (Angeli and Valanides, 2009). Usually, it is referred to as an expansion of Shulman’s (1986) concept, pedagogical content knowledge (PCK) by embedding technological knowledge (Graham, 2011; Koh and Chai, 2016). TPACK explains seven knowledge sub-domains. Content knowledge shows how educators comprehend the shreds of evidence, organizations, and degrees of content (Graham, 2011; Koh and Chai, 2016). Pedagogical knowledge shows what knowledge of overall educational values and tactics educators learn. Technological knowledge manifests educators’ technological competencies. PCK pertains to instructing particular topics with domain-specific educational approaches (Archambault and Barnett, 2010). Shulman (1986) developed PCK for the need for a theoretical structure that coherently describes the complicated nature of educator comprehension and knowledge convey. When Shulman developed PCK, he felt that academic study and policy had become too concentrated on pedagogy alone, which is damaging the content knowledge. He believed that education stakeholders mistakenly concentrate exclusively on generic educator pedagogical activities like class control, lesson planning, and organization of activity. According to Shulman questions regarding the content educators presented, the questions they posed, and the descriptions they presented were being left unanswered. Technological content knowledge (TCK) alludes to how educators consider the degree of challenges in topics or notions when making a decision about technology. Technological pedagogical knowledge (TPK) means how educators adjust overall technological and pedagogical competencies to the features of existing issues besides students’ information and frameworks.

Technological pedagogical and content knowledge is a specific frame of knowledge, i.e., it exceeds mere incorporation of the individual knowledge areas to transform knowledge of sub-areas to a specific comprehension of the values of technology for specific topics that are hardly assumed by students or are challenging to be signified by educators (Angeli and Valanides, 2009). This conversion needs both mental and metacognitive tasks required to build complex TPACK by merging information across each knowledge area (Krauskopf et al., 2015). Such high-level contemplation tasks are important since they manifest an educator’s deep comprehension of identity, choice, or integration of technology instruction objectives (Graham et al., 2012).

### Teachers’ Self-Regulated Learning

Self-regulation is usually explained as the vital aptitude of people, which is employed in different logical conditions conceded as an important part of life progress (Gestsdottir et al., 2011). Self-regulation is promising for advancing educators’ TPACK as it assists with studying and arranging their skills and assets into teaching (Chen and Jang, 2019). The fundamental presumption is that SRL is time-related and dynamic in the sense that it alters with time and in various settings (Taub et al., 2018). Through self-regulation, people can extend the capacity to understand, use and evaluate chances existing in the milieu to complete their objectives (Gestsdottir et al., 2011). Self-regulated people know how to assess their powers and weak points, supervise their professional status, make tacit efforts, and use chances and resources within the framework to achieve their purposes (Gestsdottir et al., 2010). In the educational situation, SRL refers to “a dynamic, practical procedure whereby students determine targets for their learning and try to supervise, adjust, and manage their cognitions, incentive, directed, and limited by their targets and the related characteristics within the setting (Pintrich, 2000). Self-regulated learning is a dynamic progression where the psychological capacities of learners are changed into abilities required for academic tasks. self-regulation and SRL have the core concepts in common; however, their scope is different, and in the literature, both terms are used interchangeably (Chen and Lin, 2018).

Self-regulated learning procedures in building the theories of TPACK information procedure of SRL conceptualize educator learning as a procedure of psychological model building and compatibility facilitated by meta-cognitive supervision and management procedure (Winne, 2011). This model presumes that metacognitive supervision is omnipresent during the whole regulatory procedure instead of only throughout the learning stages (Azevedo et al., 2013). Metacognitive observing could improve preservice educators’ skill of controlling their educational cycles, thereby being beneficial to their TPACK. In the preparatory stage, students can meta-cognitively supervise and assess their comprehension of the task requirements, the properness of related previous knowledge, and the development of concrete objectives and sub-objectives. Meta-cognitive procedures also guide learners, enabling them to contemplate the efficiency of research strategies, where strategies adaptively and critically change study practices for assignment objectives. Consequently, SRL is not taken into account as rigidly time-sequenced, because meta-cognition can be seen wherever it is required and builds updates as well as adaptations. In addition, studies investigating self-regulated learning delineate an extensive range of particular procedures pertaining to meta-cognition. In his study, Zimmerman (2008) examined successful self-regulated students’ conduct and specified multiple important metacognitive procedures such as preparation, self-instruction, self-supervision, and self-assessment. In this regard, Pintrich (2000) designed meta-cognitive procedures according to various learning phases, like setting objectives in the preparatory phase and thinking in the appraisal stage.

## Conclusion

Because of the technological advancement in this era, helping educators to integrate technology into education appears to be essential, but, educators have to have the related knowledge to apprehend how to implement and employ technology to change education and offer opportunities for students’ success. All educators must integrate TPACK into their practices as it is conceptualized as an advanced procedural knowledge framework. TPACK has been vastly maintained by scholars and academic practitioners as a theoretical system and instruction for educators’ expert growth (Koh and Chai, 2016). Educators, as long-lasting students, must keep expanding on their knowledge of the material, teaching technique, and technology, and figure out how to incorporate the three kinds of knowledge into their expert growth plan. Huang and Lajoie (2021) found that educators with a greater degree of TPACK showed SRL patterns with higher effectiveness, in that they did more metacognitive activities to supervise their TPACK-based lesson plan designs. Comparatively, the educators within the lower TPACK group indicated a slight attempt in supervision and assessment. Metacognitive procedures as an element of SRL provoke educators to progress toward the ultimate solution by changing their targets and tactics or looking for external help if they face challenges along the way. Self-regulated learning is presumed to have an impact on TPACK attainment, increasing educators’ comprehension of TPACK from what it is, how to execute it, and attending to real teaching assignments. The SRL framework asserts that educators’ comprehension of the intellectual requirement and assignment requirements affects their education on TPACK.

Teachers as self-regulated learners are objective-centric and have achievable objectives and sub-objective to lead educational activities and compare the profiles of presentation and results against objectives and expectations. Thus, while studying TPACK, educators must be objective-centric and the absolute objective is to leverage technology to advance learners’ education. The reviews of literature proved that computers as metacognitive instruments play the role of a stage for educators to attain SRL abilities and offer scholars a method for attaining new perceptions into the growth of teaching capabilities like course arrangement (Lajoie and Poitras, 2017) so self-regulation has a significant function in enhancing TPACK skills. People with higher self-regulation can manage their behaviors, encourage themselves and employ the thinking procedures within themselves. Primarily, self-regulation engages individuals to be constantly active and autonomous in their learning manners, and it also engages people in the social setting. It can take place when individuals tend to share the knowledge they acquire and assimilate it with their peers’ knowledge (Sukowati et al., 2020). Sell-regulation is quite significant for educators so that they could supervise learning procedures and employ diverse tactics for learning and achievement. Several activities include analyzing tasks, managing lesson materials deeply, providing details, structuring learning contents, determining learning goals, supervising results, and adapting learning tactics. Moreover, people with higher self-regulation can change the conditions of the incentive and emotive responses so that they assist their attempts and learning by employing self-talk, realistically finalizing their capabilities, and capability to learn (Edossa et al., 2019). Self-regulation in education for EFL educators is defined as an active engagement of their metacognitive, inspiration, and conduct in the educational cycle (Vinuales, 2020). Thus, educators’ self-regulation will result in active conduct in teaching and educational cycles, so that they utilize all the factors and tasks they should carry out to attain the educational objectives in technology and the TPACK element assist educators with enhancing self-regulation. It is stated that self-regulation lets educators contemplate specific methods or individual practices within each TPACK element and associate it with the other elements. Self-regulation also directs EFL educators to design and take actions to gain each element’s target. Together, self-regulation assists educators think in their decision-making about technology merging and let them adopt the link between the three important TPACK elements.

## Implications and Suggestions for Further Research

Upcoming technologies have a significant role in modern teaching and reforming the education and learning modes. This study is significant for EFL teacher educators as it assists the requirements that SRL needs to be integrated into educators’ professional education, where cognitive and meta-cognitive regulation have to turn into part of educators’ performance when they are completing educational assignments as prior studies have demonstrated that educators who have built SRL capabilities are more prepared to fulfill the requirements of the class and arrange for teaching activities (Kramarski and Michalsky, 2009). Teachers with a great level of self-regulation may be inspired to master the TPACK proficiencies due to the need to be successful in their professions. Indeed, self-regulation is significant for educators’ skills of arranging, overseeing, mirroring, responding and adjusting to ongoing transformations of the academic context (Zimmerman, 2008).

In an educational setting like EFL, self-regulated educators are defined mostly as dynamic factors that have specific academic beliefs, build proper educational procedures, manage the educational setting and situations, set targets for education and learning, plan proper course tasks, implement educational tactics, supervise and assess the efficiency of education, and modify their methods when required (Van Eekelen et al., 2005). As efficient permanent students, self-regulated educators possess the required competency to learn from education and are anticipated to use similar SRL tactics as learners, like looking for assistance from mentors, the quest for reactions and comments from learners and seeking sources useful for constant professional development (Peeters et al., 2014). Undoubtedly, developing a technology-integrated lesson needs educators to offer clear educational targets (target-setting), careful design of technological tactics to represent material and control education and learners (tactic planning), methods for developing learners’ comprehension of the material with technologies (tactic enactment), and extracts of educational performance (contemplation) that these are among the vital elements of SRL (Huang and Lajoie, 2021).

Reviewing the pertaining research is significant because gaining high-quality education in online courses needs the understanding of the methods of various fields of knowledge concerning the content, education, and technology. It significantly indicates what concepts and views prosperous educators have for subsequent education in their programs of professional development and by teaching self-regulation, one can assist educators with studying novel technologies and upgrading their TPACK appropriately. As TPACK ability is highly needed for educator trainers, it facilitates efficient education and learning along with helping successful educators to employ it effectively (Lee and Tsai, 2010). For educators, self-regulation assists them to enhance their professional knowledge and also keep their incentives (Delfino et al., 2010). It is assumed that educators with greater self-regulation competencies have a higher tendency to stimulate their knowledge development, containing their TPACK development. In addition, self-regulated educators can possess a better feeling of the particular learning and education tactics that pertain to knowledge development, also they have a higher awareness of feasible learning chances for learners and educators (Paris and Winograd, 2003).

For pre-service educators with lower education experience, various formats of SRL educational support are suggested to improve their TPCK competency. The present research clarified methods to develop professional development practices, through which in-service educators could have higher chances to investigate and run through self-regulation, and instead constantly consolidate their knowledge for education. In the situation, when one turns expert-like in TPACK functioning, the results of this research recommend that proficient educators need to be self-regulated, compatible in organizing technical tactics for education and educational difficulties, and assessing the efficiency of tactics to ensure perfect presentations. Scholars considered it greatly important to use various methods to enhance the precision of estimations of educators’ regulatory procedures in the process of TPACK development because educators can undergo the regulation procedure as a role of metacognitive practices throughout learning or problem-solving (Winne, 2011). School heads and professional development packages may offer workshops or instruction conferences to reinforce in-service educators’ knowledge and competencies of self-regulation, and simultaneously remind them to constantly use what they learned in different situations like classes and their routine lives. More studies, especially longitudinal research can be carried out in the future to provide a prospect for teacher training packages to assess how well they integrate the TPACK agenda in line with SRL to get novice educators ready to teach through technology.

## Ethics Statement

The studies involving human participants were reviewed and approved by Taiyuan University Academic Ethics Committee. The patients/participants provided their written informed consent to participate in this study.

## Author Contributions

The author confirms being the sole contributor of this work and has approved it for publication.

## Conflict of Interest

The author declares that the research was conducted in the absence of any commercial or financial relationships that could be construed as a potential conflict of interest.

## Publisher’s Note

All claims expressed in this article are solely those of the authors and do not necessarily represent those of their affiliated organizations, or those of the publisher, the editors and the reviewers. Any product that may be evaluated in this article, or claim that may be made by its manufacturer, is not guaranteed or endorsed by the publisher.
